# JAM-A overexpression is related to disease progression in diffuse large B-cell lymphoma and downregulated by lenalidomide

**DOI:** 10.1038/s41598-017-07964-5

**Published:** 2017-08-07

**Authors:** Peng-Peng Xu, Yi-Feng Sun, Ying Fang, Qi Song, Zi-Xun Yan, Yi Chen, Xu-Feng Jiang, Xiao-Chun Fei, Yan Zhao, Christophe Leboeuf, Biao Li, Chao-Fu Wang, Anne Janin, Li Wang, Wei-Li Zhao

**Affiliations:** 1 State Key Laboratory of Medical Genomics, Shanghai Institute of Hematology, Shanghai Rui Jin Hospital, Shanghai Jiao Tong University School of Medicine, Pairs, France; 2 Department of Radiology, Shanghai Rui Jin Hospital, Shanghai Jiao Tong University School of Medicine, Pairs, France; 3 Department of Nuclear Medicine, Shanghai Rui Jin Hospital, Shanghai Jiao Tong University School of Medicine, Pairs, France; 4 Department of Pathology, Shanghai Rui Jin Hospital, Shanghai Jiao Tong University School of Medicine, Pairs, France; 5 Pôle de Recherches Sino-Français en Science du Vivant et Génomique, Shanghai Rui Jin Hospital, Shanghai Jiao Tong University School of Medicine, Pairs, France; 6 U1165 Inserm/Université Paris 7, Hôpital Saint Louis, Pairs, France

## Abstract

Cancer stem cells play an important role on tumor progression. Biomarkers of stem cell property and their relationship to extranodal involvement of malignant lymphocytes are undefined in diffuse large B-cell lymphoma (DLBCL). Here we showed that junctional adhesion molecule-A (JAM-A) was highly expressed in DLBCL patients with multiple extranodal lesions. JAM-A maintained B-lymphoma cell stemness and was associated with cell invasion and epithelial-to-mesenchymal transition both *in vitro* and *in vivo*. As mechanism of action, JAM-A overexpression selectively activated transforming growth factor-β (TGF-β)/NODAL signaling, thereby enhanced B-lymphoma cell aggressiveness and induced extranodal involvement to mesoendoderm-derived organs in DLBCL. Lenalidomide downregulated JAM-A and downstream NODAL expression, resulting in inhibition of B-lymphoma cell invasion and epithelial-to-mesenchymal transition. In a murine xenograft model established with subcutaneous injection of JAM-A-overexpressing B-lymphoma cells, lenalidomide retarded tumor growth and prevented cell invasion to mesoendoderm-derived organs, consistent with the downregulation of JAM-A and NODAL expression. Collectively, these findings indicated that JAM-A was related to extranodal involvement in DLBCL through modulating TGF-β/NODAL signaling. Identified as a biomarker of stem cell property, JAM-A indicated the sensitivity of B-lymphoma cells to lenalidomide. Therapeutic targeting of JAM-A/NODAL axis could thus be a promising clinical strategy to impede tumor progression in DLBCL.

## Introduction

Diffuse large B-cell lymphoma (DLBCL), as the most common histological subtype of lymphoma, accounts for 30–40% of de novo non-Hodgkin lymphoma^1^. Being highly aggressive, DLBCL often presents the dissemination of malignant lymphocytes and subsequent formation of multiple extranodal lesions^2^, which are responsible for the vast majority of lymphoma associated deaths^3^. Recent studies have demonstrated that cancer stem cells are essential for tumor progression and thus may be potential targets for therapeutic development. Therefore, to elucidate the biomarkers of stem cell property remains of great interest for improving the clinical outcome of DLBCL patients.

Growing evidence suggests that tight junction proteins may have adhesion-independent consequences^4^. Junctional adhesion molecule-A (JAM-A) belongs to the immunoglobulin superfamily of adhesion molecules and is implicated in cell self-renewal and tumor growth^5^. JAM-A overexpression promotes cancer cell invasiveness and correlates with disease dissemination^4^. More importantly, JAM-A is highly expressed on hematopoietic stem cells with *in vivo* repopulating activity^6^. However, the role of JAM-A on lymphoma progression needs to be further investigated in DLBCL.

In the present study, we defined JAM-A as a biomarker that bridges lymphoma cell stemness with lymphoma outgrowth in mesoendoderm-derived organs via TGF-β/NODAL signaling. Therefore, therapeutic targeting of JAM-A/NODAL axis by lenalidomide represents a promising strategy to treat DLBCL.

## Results

### JAM-A was overexpressed and related to disease progression in DLBCL

**Figure 1:**
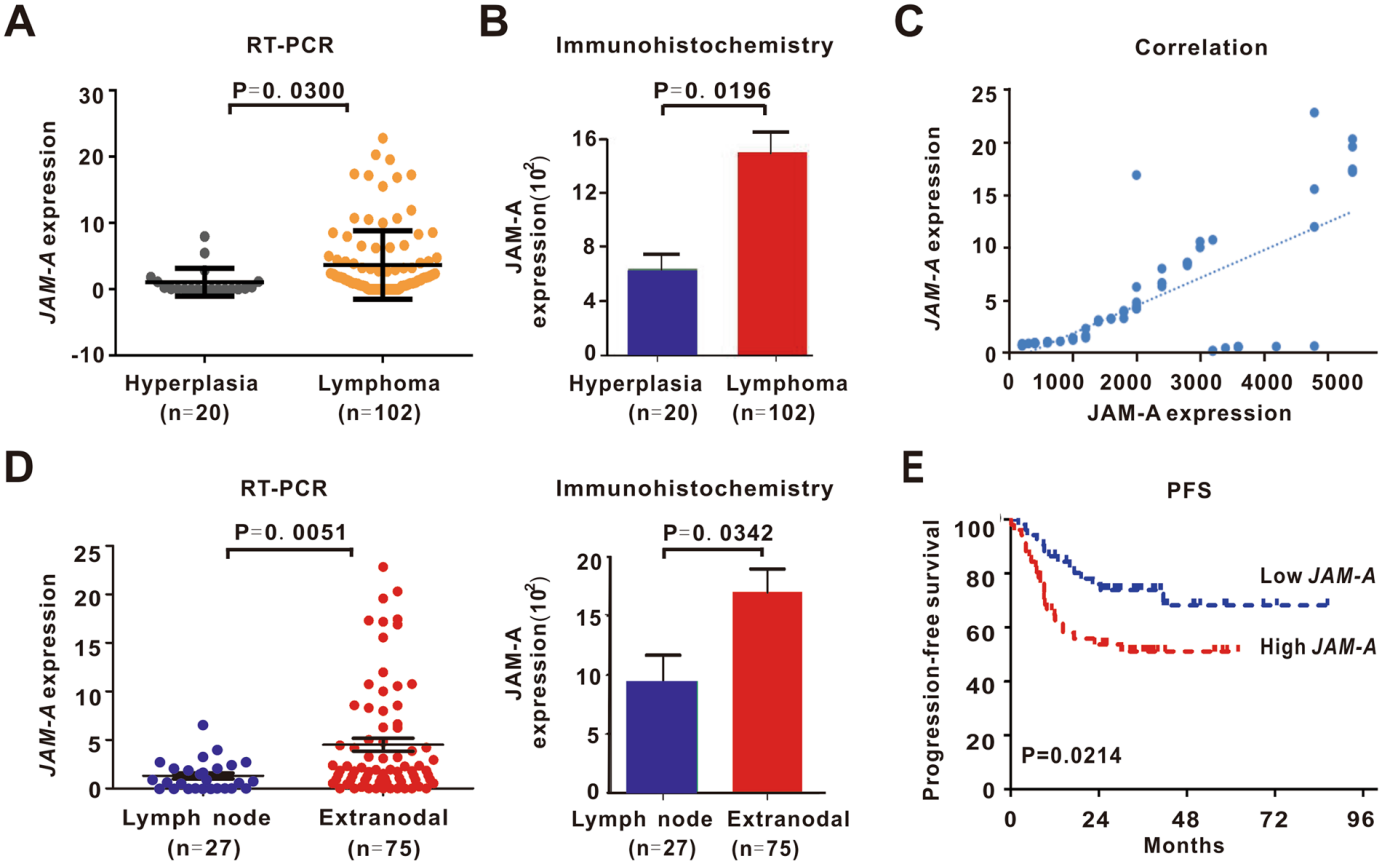
JAM-A overexpression was related to extranodal involvement and poor disease outcome in DLBCL. (**A**,**B**) *JAM-A* gene (**A**) and JAM-A protein (**B**) were overexpressed in DLBCL. (**C**) *JAM-A* gene expression correlated well with JAM-A protein expression. (**D**) DLBCL patients with extranodal involvement had higher *JAM-A* gene and JAM-A protein expression than those only with nodal lesions. (**E**) Patients in high *JAM-A* expression group had poor progression-free survival (PFS).

We first assessed JAM-A gene and protein expression in tumor samples of 102 de novo DLBCL patients using real-time quantitative RT-PCR and immunohistochemistry. JAM-A was highly expressed in DLBCL, when compared to reactive hyperplasia (N = 20) (gene expression, 3.7 ± 0.5 vs 1.1 ± 0.5, P = 0.0300, protein expression, 1501.0 ± 158.3 vs 640.0 ± 140.4, P = 0.0196, Fig. 1A,B and Supplementary Figure S1). *JAM-A* gene expression correlated well with JAM-A protein expression (Pearson Correlation Coefficient = 0.6778, P < 0.0001, Fig. 1C).

Of note, when DLBCL patients were classified in terms of involved sites, extranodal involvement group had higher expression of both *JAM-A* gene and JAM-A protein in tumor samples (P = 0.0051 and P = 0.0342, Fig. 1D). Patients with *JAM-A* level over and equal to the median value were regarded as high *JAM-A* expression, whereas those below the median value were included in the low *JAM-A* expression. Patients with high *JAM-A* expression tended to have extranodal involvement (P = 0.0421) and multiple extranodal lesions (P = 0.0294), as well as low complete remission rate (P = 0.0126, Table 1). The 2-yr progression-free survival (PFS) of patients in high *JAM-A* expression group was 53.6%, significantly shorter than those of low *JAM-A* expression group (73.8%, P = 0.0214, Fig. 1E).

**Table 1:** Clinical and biological characteristics of DLBCL patients (N = 102).

DLBCL (N = 102)	Low *JAM-A*	High *JAM-A*	P-Value
**Age**			
**>**60 years	23	23	0.8424
≤60 years	26	30	
**Sex**			
Female	20	24	0.6924
Male	29	29	
**Extranodal involvement**			
**>**1	8	19	0.0421
≤1	41	34	
**Ann Arbor Stage**			
I to II	20	22	1.0000
III to IV	29	31	
**IPI Score**			
Low to intermediate low risk (0–2)	25	29	0.8428
Intermediate high to high risk (3–5)	24	24	
**Cell of origin**			
Germinal center B-cell	18	20	1.000
Non-germinal center B-cell	30	33	
**Treatment response**			
CR	42	33	0.0126
Non-CR	7	20	
**DLBCL with extranodal lesions** (**N** = **75**)	**Low** ***JAM-A***	**High** ***JAM-A***	**P-Value**
**Extranodal involvement**			
**>**1	8	19	0.0294
=1	28	20	

### JAM-A indicated lymphoma cell stemness and epithelial-to-mesenchymal transition

**Figure 2:**
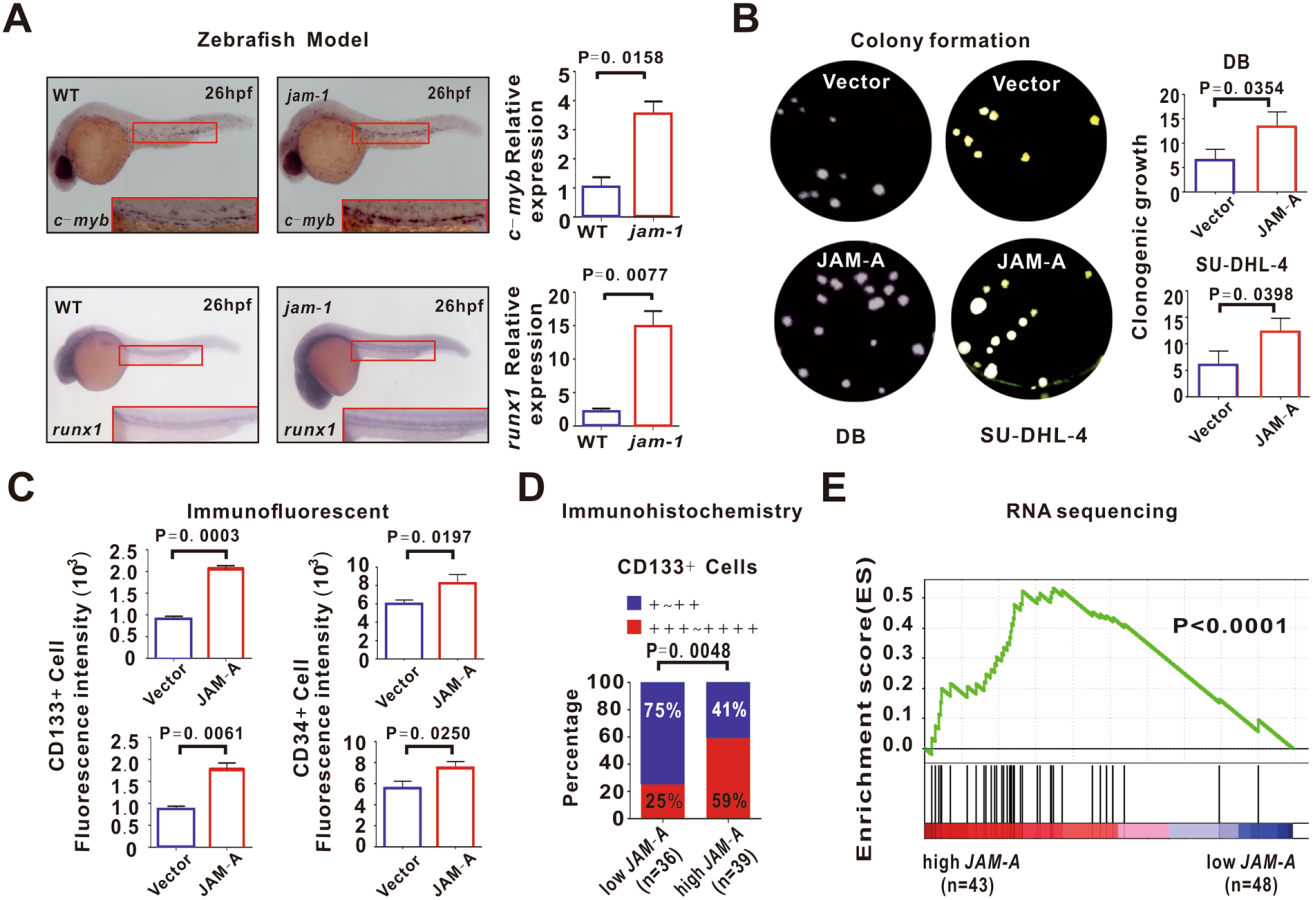
JAM-A indicated B-lymphoma cell stemness. (**A**) As assessed by Whole-mount *in Situ* Hybridization and real-time quantitative RT-PCR, *c-myb* and *runx1* were overexpressed after 26 h microinjecting *jam-1* mRNA (*jam-1*) in Zebrafish embryos. WT, wild type. (**B**,**C**) B-lymphoma cell line DB and SU-DHL-4 were transfected with control vector (Vector) and JAM-A vector (JAM-A). Ectopic expression of JAM-A was associated with increased colony formation (**B**) and stem-cell marker CD133 and CD34 expression (**C**). (**D**) DLBCL patients with high *JAM-A* expression displayed increased CD133 positivity. (**E**) Patients in high *JAM-A* expression group were enriched for a stem cell gene signature, as revealed by RNA sequencing. Data in (**A**), (**B**) and (**C**) are representative of three independent experiments.

To gain insights into the biological function of JAM-A, we established a JAM-A-transgenic zebrafish model by microinjecting *jam-1* (zebrafish JAM-A gene identified in genome data base UCSC) mRNA into zebrafish embryos. Jam-1 overexpression led to a significant elevation of zebrafish hematopoietic stem-cell markers *c-myb* and *runx1* ^7^, when compared to a wild-type (WT) zebrafish (P = 0.0158 and P = 0.0077, Fig. 2A). We also transfected B-lymphoma cell line DB and SU-DHL-4 with JAM-A vector (JAM-A). Ectopic expression of JAM-A stimulated lymphoma cell colony formation (P = 0.0354 and P = 0.0398, Fig. 2B) and induced higher expression of cancer stem-cell marker CD133 and CD34 than those of control vector (Vector) (P = 0.0003 and P = 0.0061 for CD133, P = 0.0197 and P = 0.0250 for CD34, Fig. 2C). Clinically, DLBCL patients with high *JAM-A* expression displayed a remarkable increase in tumor CD133 positivity (+++~++++) than those of low *JAM-A* expression (P = 0.0048, Fig. 2D). Interestingly, high JAM-A group was enriched for a stem cell gene signature, as revealed by RNA-sequencing (P < 0.0001, Fig. 2E).

**Figure 3:**
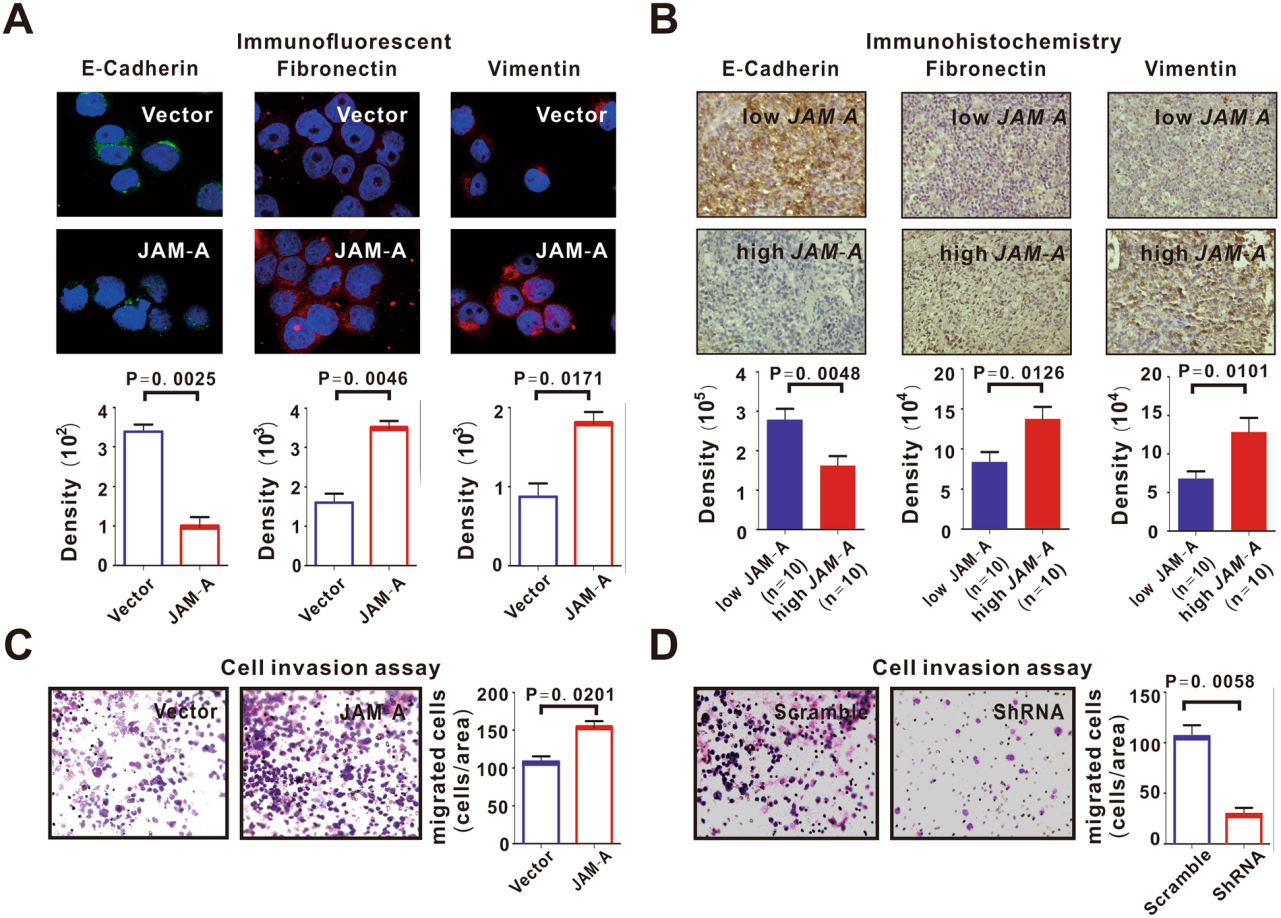
JAM-A induced B-lymphoma cell invasion. (**A**,**B**) Epithelial-mesenchymal transition (EMT) was observed in JAM-A-overexpressing DB cells (**A**) and in DLBCL patients with high *JAM-A* expression (**B**). (**C**,**D**) JAM-A-transfected DB cells acquired increased cell invasion (**C**), which was inhibited in JAM-A-ShRNA-transfected cells (**D**). Data in (**A**,**C** and **D**) are representative of three independent experiments.

Moreover, stem cells undergo a process known as epithelial-to-mesenchymal transition (EMT)^8^, defined by downregulated epithelial marker E-Cadherin with upregulated mesenchymal markers Fibronectin and Vimentin^9^. Indeed, EMT was observed in both JAM-A-transfected B-lymphoma cells (P = 0.0025, P = 0.0046 and P = 0.0171, Fig. 3A) and the tumor samples of DLBCL patients with high *JAM-A* expression (P = 0.0048, P = 0.0126 and P = 0.0101, Fig. 3B). As revealed by cell invasion assay, B-lymphoma cells with JAM-A overexpression achieved a notably higher percentage of cell invasion than those transfected with control vector (P = 0.0201, Fig. 3C). As compared to scramble cells, this invasive ability was inhibited by the molecular silencing of JAM-A using ShRNA (P = 0.0058, Fig. 3D). Thereafter, cancer cells required stemness to potentially reach out distant organs and initiate tumor metastasis^10^.

### JAM-A induced lymphoma cell invasion to mesoendoderm-derived extranodal organs via TGF-β/NODAL signaling

**Figure 4:**
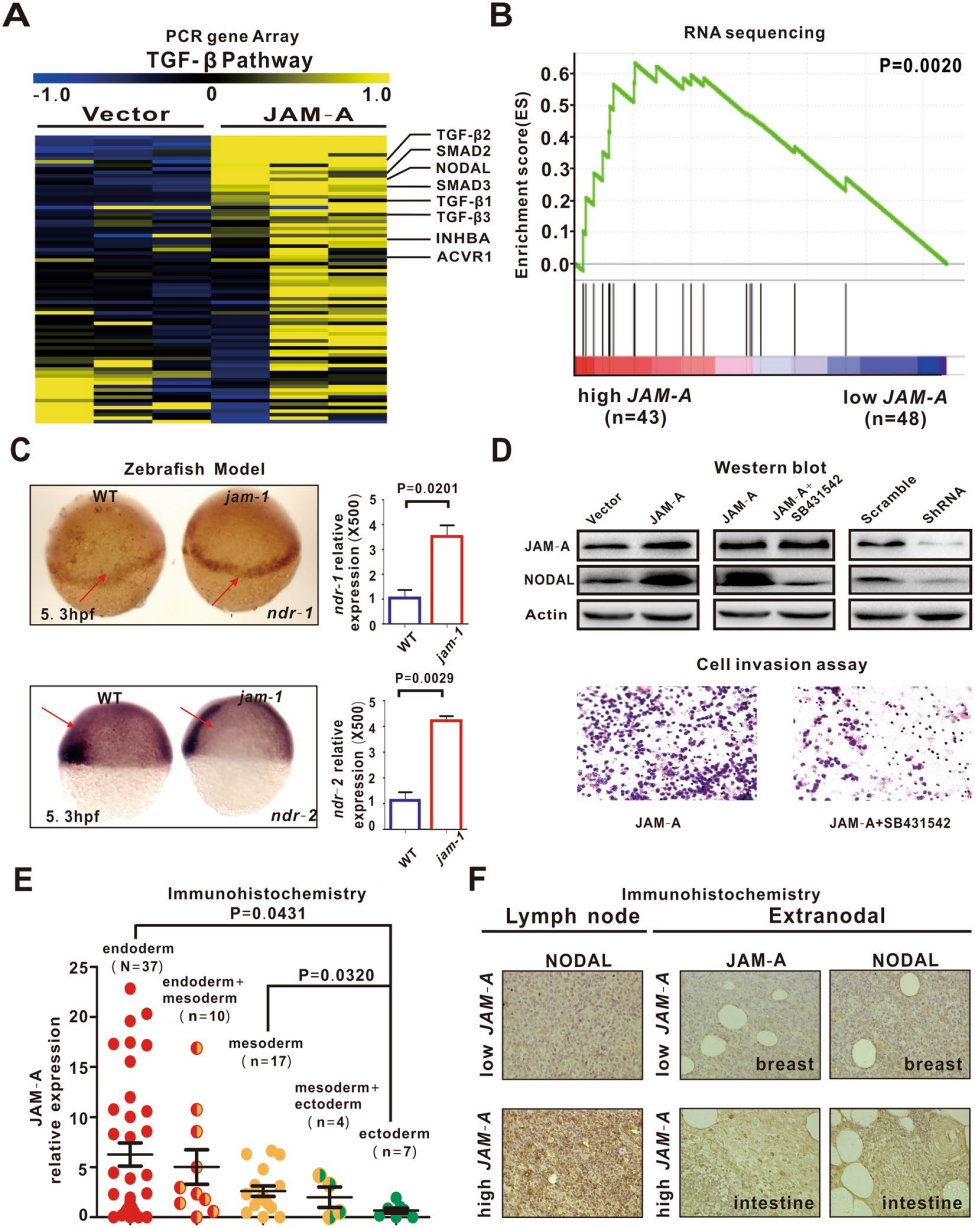
JAM-A activated TGF-β/NODAL signaling. (**A**,**B**) In JAM-A-transfected DB cells (**A**) and DLBCL patients (**B**), JAM-A overexpression was related to activation of TGF-β/NODAL signaling. (**C**) As assessed by Whole-mount *in Situ* Hybridization and real-time quantitative RT-PCR, *ndr-1* and *ndr-2* were increased in jam-1-overexpressing zebrafish. WT, wild type. (**D**) JAM-A upregulated NODAL expression, which was restored by specific TGF-β/NODAL/Smad inhibitor SB431542 or JAM-A ShRNA. SB431542 abrogated JAM-A-induced lymphoma cell invasion (**E**) JAM-A was the highest in patients with involvement of endoderm-derived organs, followed by those with involvement of mesoderm- and ectoderm-derived organs. (**F**) JAM-A correlated with increased NODAL expression in primary lymph nodes, as well as in secondary lesions. Data in (**C**) are representative of three independent experiments.

The EMT is triggered by many extracellular signals in cancer cells and the most potent inducers are members of the TGF-β family. As revealed by PCR gene array and RNA sequencing, TGF-β signaling pathway was activated both in JAM-A-transfected B-lymphoma cells (Fig. 4A) and in DLBCL patients (Fig. 4B). NODAL, an important member of TGF-β family proteins, is involved in embryonic stem cell maintenance and differentiation^11^. Moreover, in zebrafish models, key markers of NODAL signaling, *ndr-1* (squint) and *ndr-2* (cyclops), were higher in jam-1-overexpressing group than in WT group (P = 0.0201 and P = 0.0029, Fig. 4C). In B-lymphoma cells, transfection of JAM-A increased NODAL expression, while treatment with specific TGF-β/NODAL/Smad inhibitor SB431542 abrogated JAM-A-induced NODAL upregulation and lymphoma cell invasion (Fig. 4D and Supplementary Figure S3A). Also, molecular silencing of JAM-A by ShRNA counteracted JAM-A-induced NODAL expression (Fig. 4D and Supplementary Figure S3A). Together, these data confirmed that JAM-A activated TGF-β/NODAL signaling.

Interestingly, according to the germinal layers that derived from extranodal lesions in DLBCL patients, JAM-A overexpression was associated with mesoendoderm-derived organs (intestine, n = 15, stomach, n = 11, liver, n = 7, bone, n = 5, lung, n = 4, etc.), instead of ectoderm-derived organs (breast, n = 4, sensory organs, n = 3, etc.) (endoderm vs ectoderm, P = 0.0431, mesoderm vs ectoderm, P = 0.0320, Fig. 4E). Meanwhile, in high *JAM-A* expression group, JAM-A coexist with increased NODAL expression, both in primary and in secondary lesions of endoderm- and mesoderm-associated organs (Fig. 4F).

### Lenalidomide modulated JAM-A/NODAL axis and impeded metastatic lymphoma outgrowth to mesoendoderm-derived organs

**Figure 5:**
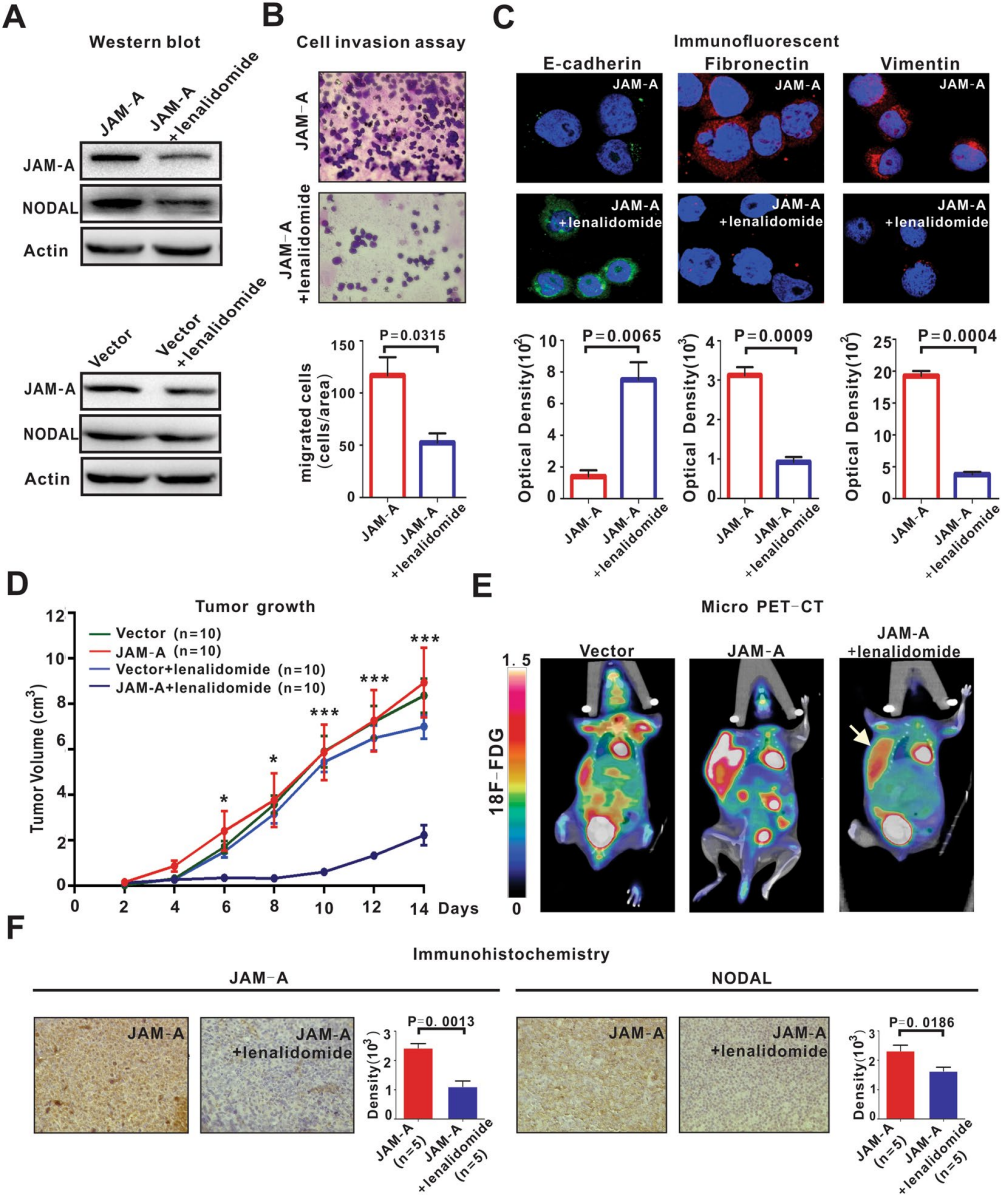
Lenalidomide inhibited lymphoma progression. (**A**) Lenalidomide (1 μM) downregulated JAM-A and NODAL expression more significantly in JAM-A-transfected DB cells as compared to vector-transfected cells. (**B**,**C**) Lenalidomide (1 μM) inhibited JAM-A-transfected cell invasion (**B**) and EMT (**C**). (**D**) In murine xenograft model established with subcutaneous injection of DB cells, tumor size was similar between vector-transfected (Vector) and JAM-A-transfected (JAM-A) group. Lenalidomide (25 mg/kg/day, JAM-A + lenalidomide) retarded tumor growth. *P < 0.05 and ***P < 0.001 comparing with the JAM-A group. (**E**) By micro-PET-CT, JAM-A overexpression contributed to tumor metastasis, all involving endoderm- and mesoderm-associated organs, which was inhibited by lenalidomide. (**F**) Lenalidomide downregulated JAM-A and NODAL expression. Data in (**B**) and (**C**) are representative of three independent experiments.

Lenalidomide is effective in treating hematological malignancies including lymphoma^12^. Lenalidomide downregulated JAM-A and NODAL expression more significantly in JAM-A-transfected B-lymphoma cells, as compared to vector-transfected cells (Fig. 5A and Supplementary Figure S3), consistent with inhibition of cell invasion (P = 0.0315, Fig. 5B) and EMT (P = 0.0065, P = 0.0009 and P = 0.0004, Fig. 5C). To further clarify how lenalidomide reduced *JAM-A* expression, the stability of *JAM-A* mRNA was estimated by measuring mRNA levels after treatment with Actinomycin D (5 μg/ml), a nucleic acid synthesis inhibitor, for the indicated times by quantitative real-time PCR. The results showed that lenalidomide decreases the transcript stability and promotes the degradation of *JAM-A* mRNA (Supplementary Figure S2).

Meanwhile, we established a murine xenograft model with a subcutaneous injection of JAM-A-transfected B-lymphoma cells. With similar growth of tumors formed by vector-transfected cells (Fig. 5D), JAM-A-overexpressing cells were responsible for higher frequency of tumor metastasis (Fig. 5E). The metastatic lesions revealed by PET-CT all belonged to endoderm- and mesoderm-derived organs (intestine, n = 2, stomach, n = 1 and liver, n = 1, representative images shown in Fig. 5E). Compared to the untreated mice, tumor size remarkably decreased in lenalidomide-treated high JAM-A expression group and lymphoma invasion was significantly reduced (Fig. 5D,E). In accordance with *in vitro* data, the expression of JAM-A and NODAL was prohibited in the lenalidomide-treated high JAM-A expression group (P = 0.0013 and P = 0.0186, Fig. 5F).

## Discussion

Disease dissemination, presented by multiple extranodal involvement, is an essential adverse prognostic factor of DLBCL patients. Biomarkers related to extranodal involvement are not well determined in lymphoma. Here we showed that JAM-A overexpression is associated with multiple extranodal lesions and poor disease outcome with lower CR rate and shorter PFS. In addition to solid tumors^5^, our results provided a direct link of JAM-A to disease progression in DLBCL.

Recent data indicate that non-stem cancer cells can re-enter the stem state and the metastatic disease in patients is concomitant with the appearance of stemness and EMT in these cells^13^. Overexpressed on hematopoietic stem cells with *in vivo* repopulating activity^6^ and subsequently identified as a cancer stem-cell maintenance factor^14^, JAM-A may reprogram lymphoma cells to a de-differentiated stem-like state, contributing to lymphoma progression in DLBCL.

NODAL signaling is not only responsible for the specification of endoderm and mesoderm during embryogenesis^15^, but also re-emerges during cancer development^16–18^. Mimicking embryogenic development, NODAL stimulates cancer cell growth and promotes metastasis in solid tumors^19^. Overexpression of NODAL in breast cancer induces ectoderm-derived breast tissue to mesoderm-derived lymph nodes^20^. Also, JAM-A is upregulated in mesoendoderm-associated tumors like non-small cell lung cancer and gastric cancer^21, 22^. In the present study, JAM-A may facilitate lymphoma progression in DLBCL via TGF-β/NODAL signaling, committing cell invasion towards endoderm- and mesoderm-derived extranodal organs. However, the molecular mechanism of action needs to be further investigated.

Lenalidomide is an oral immunomodulatory drug with direct and indirect antineoplastic activity for aggressive or indolent B-cell lymphoma^23^. With the indirect effect mediating through multiple types of immune cells within the tumor microenvironment, recent studies have identified molecular targets that exert the direct effect of lenalidomide on cancer cells, including adhesion molecules like intercellular cell adhesion molecule-1 and vascular cell adhesion molecule-1^24, 25^. Our data confirmed JAM-A as an important adhesion molecule and also as a potential therapeutic target of lenalidomide.

## Conclusions

JAM-A is an unfavorable prognostic biomarker related to stem cell property in DLBCL and contributed to extranodal commitment to mesoendoderm-derived organs through the activation of TGF-β/NODAL signaling. JAM-A indicated the sensitivity of B-lymphoma cells to lenalidomide, and therapeutic targeting of JAM-A/NODAL axis represents a promising clinical strategy to counteract tumor progression in DLBCL.

## Methods

### Patients

One-hundred and two patients diagnosed as DLBCL were included in this study. The histological diagnosis was established according to WHO classification^26^. All the patients were CD20 positive DLBCL with exclusion of mediastinal large B-cell lymphoma. Clinical characteristics of the patients were listed in Table 1. Twenty age- and sex-matched cases with reactive hyperplasia were referred as controls. The study was approved by Shanghai Rui Jin Hospital Review Board and written informed consent were obtained from patients in accordance with the Declaration of Helsinki. All experimental protocols were performed in accordance with relevant guidelines.

### Cell lines and reagents

B-lymphoma cell line DB and SU-DHL-4 were available from American Type Culture Collection (ATCC, Manassas, VA, USA). Cells were cultured in RPMI-1640 medium with 10% heat-inactivated fetal bovine serum (FBS) in a humidified atmosphere of 95% air and 5% CO_2_ at 37 °C. Specific TGF-β/NODAL/Smad inhibitor SB431542 was from Sigma (St. Louis, MO, USA). Lenalidomide was from Cayman Chemical (Ann Arbor, MI, USA). Nucleic acid synthesis inhibitor Actinomycin was from Abcam (Cambridge, UK).

### Colony formation assay

Cells were mixed with an equal volume of 0.7% soft agarose and then plated in 6-well plates with 3500 cells per well for 21 days. Colony formation was calculated as count of colonies with a diameter of more than 0.1 mm divided by total number of cells^27^.

### Flow cytometry

Surface CD133 and CD34 were analyzed using anti-CD133-PE and anti -CD34-PE (Miltenyi Biotec, Bergisch-Gladbach, Germany), as previously described^28^.

### Real-time quantitative RT-PCR

Total RNA was extracted from frozen sections using Trizol agent (Invitrogen, Carlsbad, CA, USA) and cDNA was reverse transcribed by the reverse transcription kit (TAKARA, Japan). JAM-A expression was analyzed by real-time quantitative RT-PCR using 7500HT Fast Real-time PCR system (Applied Biosystem, Foster City, CA, USA). *JAM-A*: Forward, 5′-GTGCCTTCAGCAACTCTTCC-3′ and Reverse, 5′-ACCAGATGCCAAAAACCAAG-3′. *GAPDH*: Forward, 5′-GAAGGTGAAGGTCGGAGTC-3′ and Reverse, 5′-GAAGATGGTGATGGGATTTC-3. DB and SU-DHL-4 cells were used for calibration. Zebrafish *c-myb*: Forward, 5′-AACAACGGCAACAGAAGTGC-3′ and Reverse, 5′-TTGGGAGTTCGGAACAGCTC-3′. Zebrafish *runx1*: Forward, 5′-CCTGGTCGTATGAGCAGTCG-3′ and Reverse, 5′-GAAACGCCCATCTGGGAGAG-3′. Zebrafish *ndr-1*: Forward, 5′-ACTGGTTGCACCAGAGTGAG-3′ and Reverse, 5′-ACATACTTGGAGTGCTCGGC-3′. Zebrafish *ndr-2*: Forward, 5′-GAGCTGCAGAGAACACCACT-3′ and Reverse, 5′- CAGGATGCAGGAACACGACT-3′. Zebrafish *actin*: Forward, 5′-GCCGTGACCTGACTGACTACCT-3′ and Reverse, 5′-CGCAAGATTCCATACCCAAGA-3′. Relative expressions were calculated by the method of ΔΔCT.

### Cell transfection

Lenti-virus with *JAM-A* or control vector and *JAM-A* ShRNA or scramble vector for cell transfection were synthesized by GENECHEM (Shanghai, China). DB and SU-DHL-4 cells were infected by lenti-virus with cell-virus ratio of 1:100 according to the manufacturer’s protocol. The stably transfected clones were selected by green fluorescence protein 72 hours after infection.

### Western blot

Western blot was performed as described previously^28^. Antibody against JAM-A was from Life Technologies (Gaithersburg, MD, USA). Antibody against NODAL was from Abcam (Cambridge, MA, USA). Actin (Cell Signaling technology, Beverly, MA, USA) was used to ensure equivalent protein loading.

### Immunohistochemistry and Immunofluorescent assay

Immunohistochemical analyses were performed on 5μm paraffin sections with an indirect immunoperoxidase method by antibodies against JAM-A (Life Technologies, 1:100 dilution), E-Cadherin, Vimentin, Fibronectin (Affinity biosciences, Piscataway, NJ, USA, 1:200 dilution) and NODAL (Abcam, 1:200 dilution). Immunofluorescent assays were performed on acetone-fixed cells with rabbit anti-human-E-Cadherin, Vimentin and Fibronectin as primary antibodies, and Alexa Fluor-conjugated donkey anti-mouse-IgG and anti-rabbit-IgG antibodies (Invitrogen, Carlsbad, CA, USA) as secondary antibodies. Nuclei were stained by DAPI.

The protein expression levels were scored based on staining intensity (SI) and distribution using the immunoreactive score (IRS). Briefly, IRS = SI × PA (positive area). The intensity of immunohistochemical and immunofluorescence staining was measured by the Image-Pro Plus 6.0 image analysis software (Media Cybernetics, Inc., Silver Spring, USA)^29^.

### PCR gene Array

TGF-β PCR gene Array (SABiosciences/Qiagen, Frederick, MD, USA) was performed by 7500HT Fast Real-time PCR system according to the manufacturer’s instructions.

### RNA sequencing

RNA was extracted using Trizol and RNeasy MinElute Cleanup kit from tissue samples and using PAXgene Blood miRNA kit from blood samples. Globin RNA was removed using Globin-Zero Gold rRNA Removal kit for RNA from blood samples. Following extraction, the RNA quantity was evaluated on Nanodrop and the integrity of total RNA using RNA 6000 Nano kit on Aligent 2100 Bioanalyzer. RNA library was constructed using TruSeq RNA Sample Preparation kit. The poly-A containing mRNA molecules was purified using oligo-dT attached magnetic beads. Following purification, the mRNA was fragmented into small pieces using divalent cations under elevated temperature. The cleaved RNA fragments were copied into first strand cDNA using reverse transcriptase and random primers, followed by second strand cDNA synthesis using DNA Polymerase I and RNase H. The cDNA fragments went through an end repair process, the addition of a single ‘A’ base, and ligation of the adapters. The products were purified and enriched with PCR to create the final cDNA library. The clusters of the cDNA library were generated on the flow cell using TruSeq PE Cluster kit and HiSeq PE flow cell. The clusters were finally sequenced on HiSeq. 2000 system using TruSeq SBS kit.

### Cloning and plasmid construction in Zebrafish

Adult zebrafish (Danio rerio) were raised following established protocols. Zebrafish *jam-1* gene was identified according to homology to human JAM-A. The specific primers were designed based on genomic sequence in the UCSC data base (University of California, Santa Cruz) to amplify part of *jam-1* gene. The plasmid was constructed by GENECHEM, Shanghai, China. Animals were used according to the protocols approved by the Shanghai Rui Jin Hospital Animal Care and Use Committee.

### Extraction of mRNA and microinjection of Zebrafish embryos

mRNA of *jam-1* was extracted and diluted to DEPC water at the concentration of 100 ng/μl. mRNA were microinjected at a volume of 2 nl into one-cell stage embryos using an air pressure injector and glass capillaries. Injection experiments were performed in triplicate.

### Whole-mount *in Situ* Hybridization in Zebrafish

pCS2+containing part of *c-myb*, *ndr-1* or *ndr-2* was used to produce antisense RNA probes using digoxigenin-11-uridine 59-triphosphate. Zebrafish embryos were fixed in 4% paraformaldehyde at the stages indicated and Whole-mount *in Situ* Hybridization was performed following the manufacturer’s protocol.

### Cell invasion assay

Cell invasion was tested in the Matrigel Invasion Chamber (BD Pharmingen, Franklin Lakes, NJ, USA), composed of the upper and lower compartment separated by the polycarbonate membranes (8 μm pore size). The upper side of the membrane was coated with Matrigel matrix. 6 × 10^4^ cells were incubated with RPMI-1640 (FBS-free, 200 μl) for 24 hours and added to the upper compartment, while RPMI-1640 with 10% FBS (500 μl) was added to the lower compartment. After incubation with 5% CO_2_ at 37 °C for 24 hours, the non-migratory cells remained on the Matrigel matrix were removed and the membrane was stained by Wright-Giemsa staining. Migratory cells were observed under the microscope at 40× magnifications and counted in different fields of membranes in triplicate.

### Murine model

Severe combined immune deficiency mice (5–6-week-old) were obtained from Shanghai Laboratory Animal Center (Shanghai, China) and injected subcutaneously into the right flank with 2 × 10^7^ DB cells transfected with JAM-A vector (JAM-A group) or control vector (control group, 10 mice per group). Tumor volumes were calculated as 0.5 × a × b^2^, where ‘a’ is the length and ‘b’ is the width. Treatments (10 mice per group) were started after the tumor became about 0.5 × 0.5 cm in surface (day 0). The JAM-A group received distilled water, whereas the JAM-A + lenalidomide group received lenalidomide (25 mg/kg/day) orally for two weeks. Animals were used according to the protocols approved by the Shanghai Rui Jin Hospital Animal Care and Use Committee.

### Micro-PET/CT imaging

Mice were put to the test of positron emission tomography-computed tomography (PET/CT) after two weeks of treatment. Through the tail vein of anesthetized mice, 18F-fluorodeoxyglucose (18F-FDG, 0.1 ml per injection with an activity of 5.5 MBq) was injected. PET/CT imaging was performed on an Inveon MM Platform (Siemens Preclinical Solutions, Knoxville, TN, USA) with 8.5 cm transaxial and 5.7 cm axial fields of view, as previously described^30^.

### Statistical analysis

Differences of JAM-A expression among groups were assessed by the Mann-Whitney U test. *In vitro* experimental results were expressed as mean ± SEM. of data obtained from three separate experiments and determined using a t-test to compare variance. Chi-square test was used when comparing the constitution ratio between two groups. P < 0.05 was considered statistically significant.

### Data Availability

All data generated or analyzed during this study are included in this published article (and its Supplementary Information files).

## Electronic supplementary material

Supplementary Info
