# Supplementary material for: JAM-A overexpression is related to disease progression in diffuse large B-cell lymphoma and downregulated by lenalidomide
Source: Sci Rep. 2017 Aug 7;7:7433. doi: 10.1038/s41598-017-07964-5 (PMC5547054; doi:10.1038/s41598-017-07964-5)
Supplement: Supplementary file 1 — Supplementary Info [file 41598_2017_7964_MOESM1_ESM.doc]

**JAM-A overexpression is related to disease progression in diffuse large B-cell lymphoma and downregulated by lenalidomide**

Peng-Peng Xu^1*^, Yi-Feng Sun^1*^, Ying Fang^1*^, Qi Song^2*^, Zi-Xun Yan^1^, Yi Chen^1^, Xu-Feng Jiang^3^, Xiao-Chun Fei^4^, Yan Zhao^1^, Christophe Leboeuf^5,6^, Biao Li^3^, Chao-Fu Wang^4^, Anne Janin^5,6^, Li Wang^1,5†^, Wei-Li Zhao^1,5†^

^1^ State Key Laboratory of Medical Genomics, Shanghai Institute of Hematology, Shanghai Rui Jin Hospital, Shanghai Jiao Tong University School of Medicine;

^2^ Department of Radiology, Shanghai Rui Jin Hospital, Shanghai Jiao Tong University School of Medicine;

^3^ Department of Nuclear Medicine, Shanghai Rui Jin Hospital, Shanghai Jiao Tong University School of Medicine;

^4^ Department of Pathology, Shanghai Rui Jin Hospital, Shanghai Jiao Tong University School of Medicine;

^5^ Pôle de Recherches Sino-Français en Science du Vivant et Génomique, Shanghai Rui Jin Hospital, Shanghai Jiao Tong University School of Medicine;

^6^ U1165 Inserm/Université Paris 7, Hôpital Saint Louis, Pairs, France.

* These authors contributed equally to this work.

† These authors contributed equally to this work.

Correspondence to: Wei-Li Zhao, Email: [zhaoweili_sih@163.com](mailto:zhaoweili_sih@163.com) and Li Wang, Email: wl_wangdong@126.com; State Key Laboratory of Medical Genomics; Shanghai Institute of Hematology; Shanghai Rui Jin Hospital; Shanghai, China; Tel: 0086-21-64370045; Fax: 0086-21-64743206.


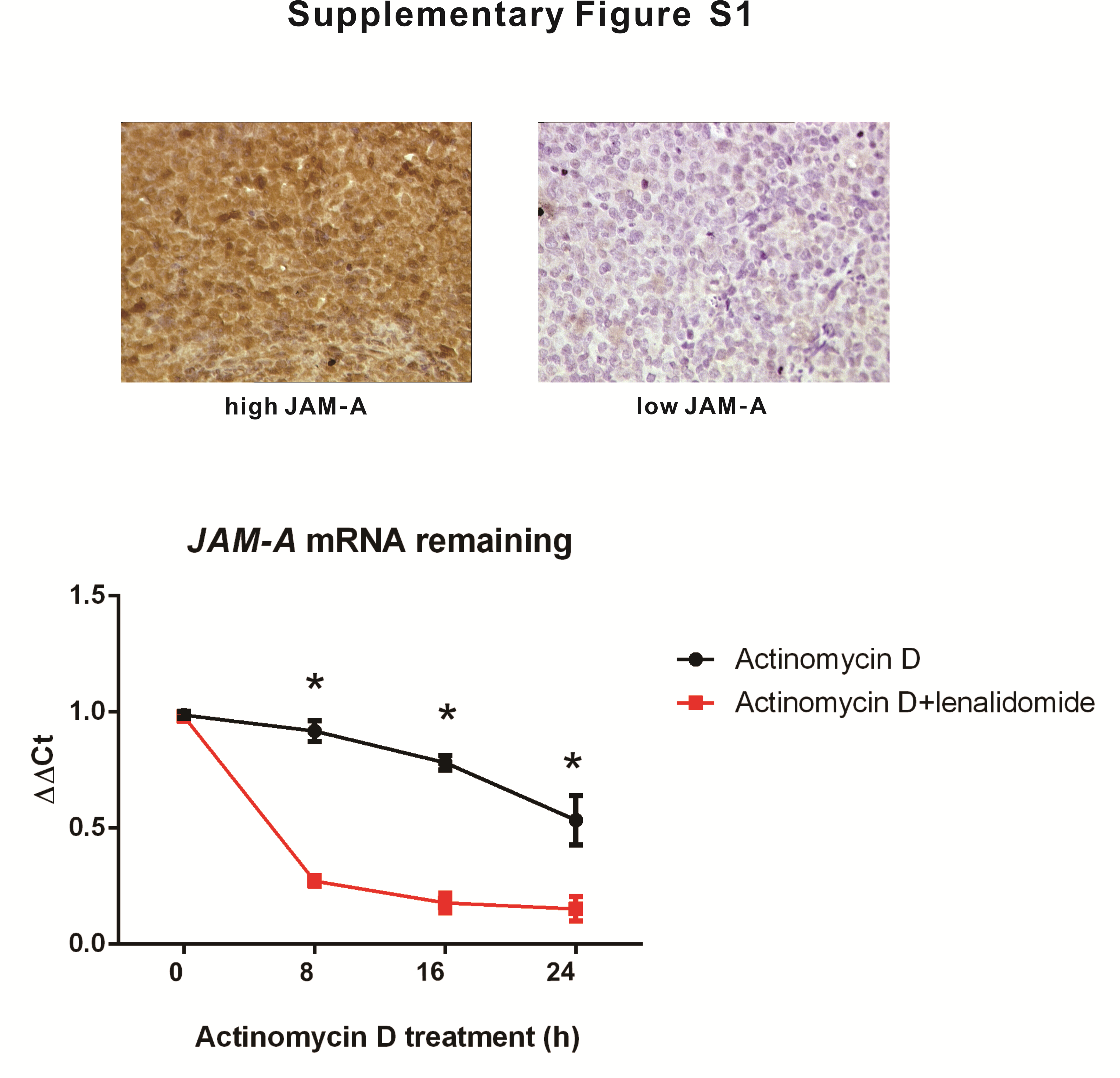
**Supplementary Fig. S1 Representative images of immunohistochemistry staining from tumor samples of DLBCL patients with high JAM-A and with low JAM-A expression.**

**
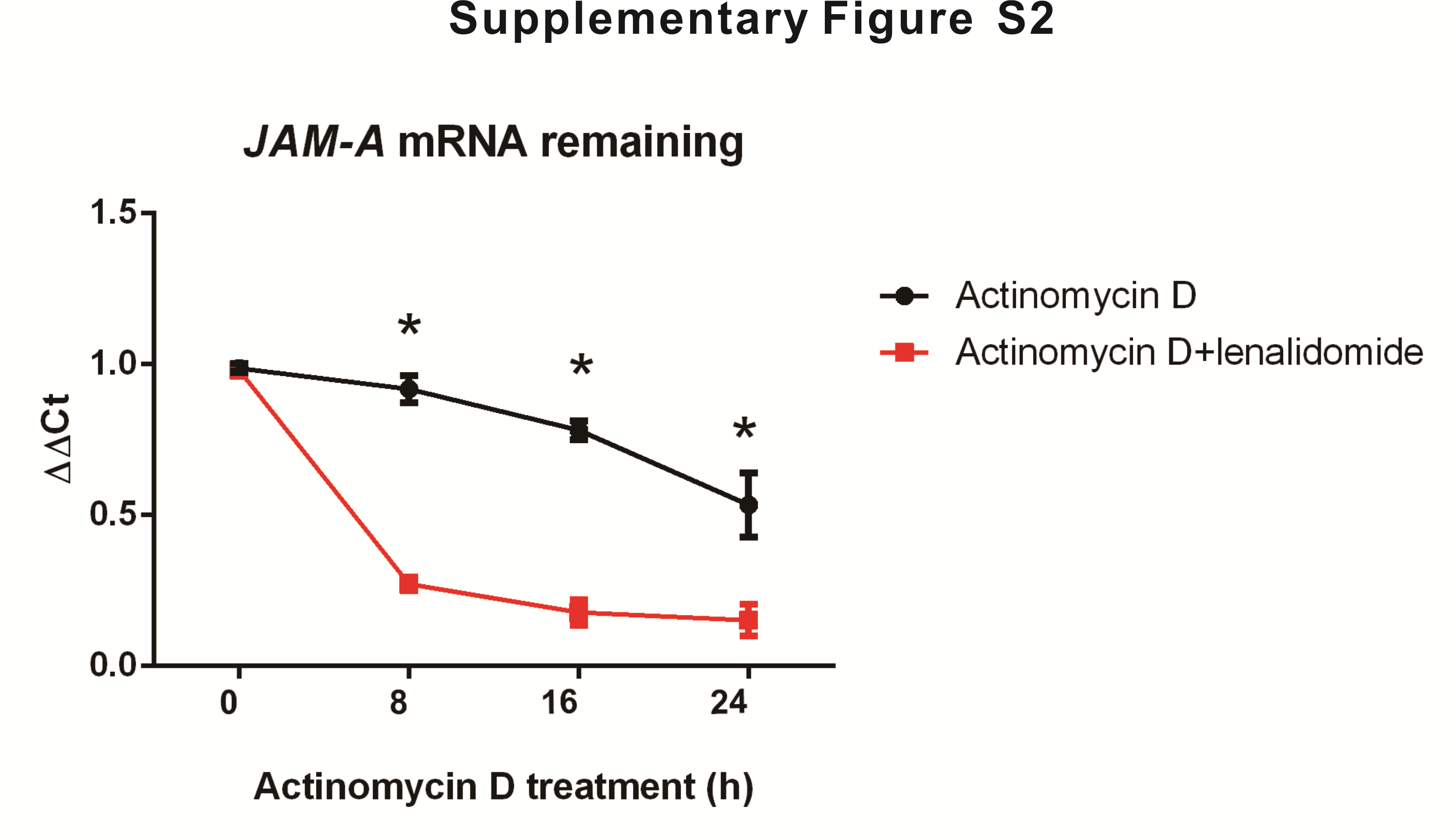
Supplementary Fig. S2 Lenalidomide decreased the transcript stability of *JAM-A* mRNA in B-lymphoma cells.**

Actinomycin D (5μg/ml) was added to JAM-A-transfected DB cells for different intervals, either alone or treated with lenalidomide (1μM) for 24h, DB cells treated with Actinomycin D and lenalidomide had lower *JAM-A* mRNA remaining compared with those treated with Actinomycin D alone.


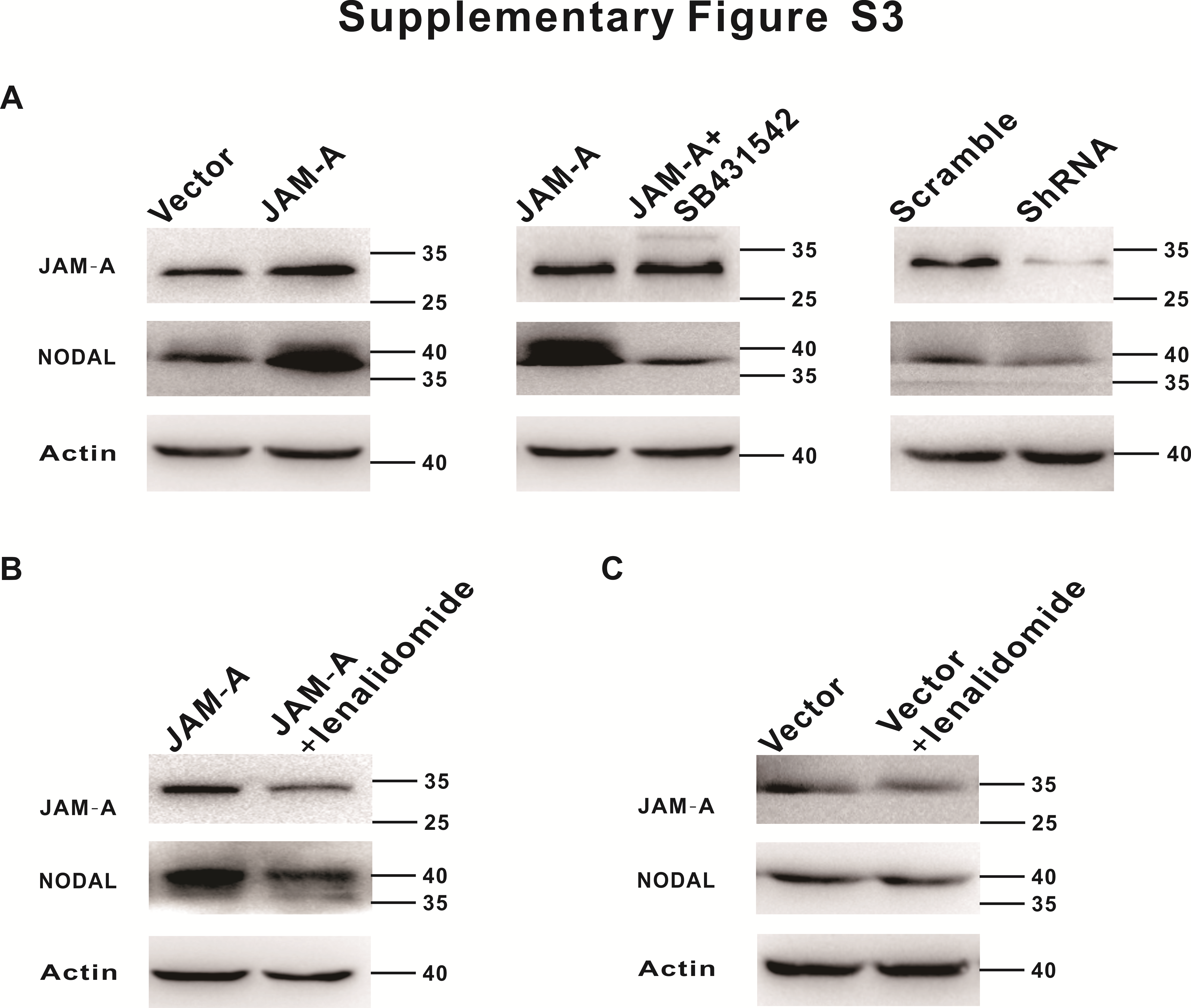


**Supplementary Fig. S3 Full size blots of the immunoblot detection.**

A: Full size blots of the immunoblot detection shown in Figure 4D. B and C: Full size blots of the immunoblot detection shown in Figure 5A.
